# Is Interleukin-6 a better predictor of successful antibiotic therapy than procalcitonin and C-reactive protein? A single center study in critically ill adults

**DOI:** 10.1186/s12879-019-3800-2

**Published:** 2019-02-13

**Authors:** Lorenz Weidhase, Daniel Wellhöfer, Gero Schulze, Thorsten Kaiser, Tim Drogies, Ulrike Wurst, Sirak Petros

**Affiliations:** 1 Medical Intensive Care Unit, University Hospital of Leipzig, Liebigstraße 20, 04103 Leipzig, Germany; 2 Carl-von-Basedow-Klinikum Merseburg, Medical Clinic I, Weiße Mauer 52, 06217 Merseburg, Germany; 3 Department of Internal Medicine, Helios Hospital Schkeuditz, Leipziger Straße 45, 04435 Schkeuditz, Germany; 4 Institute of Laboratory Medicine, Clinical Chemistry and Molecular Diagnostics, University Hospital Leipzig, Paul-List-Straße 13/15, 04103 Leipzig, Germany; 5 Medical central laboratory Altenburg, Am Waldessaum 8, 04600 Altenburg, Germany; 6 University Hospital of Leipzig, Center for Pediatric Research Leipzig, University Hospital for Children &Adolescents, Liebigstraße 20, 04103 Leipzig, Germany; 7 Medical Intensive Care Unit, University Hospital of Leipzig, Liebigstraße 20, 04103 Leipzig, Germany

**Keywords:** Sepsis, Interleukin-6 (IL-6), Procalcitonin (PCT), C-reactive protein (CRP), Antibiotic therapy, Prognosis

## Abstract

**Background:**

The aim of this study was to evaluate whether Interleukin-6 (IL-6) could be a faster indicator of treatment success in adults with severe sepsis and septic shock compared to procalcitonin (PCT) and C-reactive protein (CRP).

**Methods:**

Data from adult patients with severe sepsis and septic shock managed at the medical intensive care unit (ICU) of the University Hospital Leipzig between September 2009 and January 2012 were analyzed retrospectively. Values for CRP, PCT and IL-6 on admission as well as after 24 and 48–72 h were collected. Antibiotic therapy was defined as clinically successful if the patient survived ICU stay.

**Results:**

A total of 328 patients with severe sepsis and septic shock with adequate data quality were included. After 48–72 h, the median IL-6 was significantly lower in survivors than in non-survivors (114.2 pg/ml vs. 746.6 pg/ml; *p* < 0.001), while there was no significant difference for PCT (5.6 vs. 4.9 ng/ml; *p* = 0.586) and CRP (158.5 mg/l vs. 172.4 mg/l; *p* = 0.988).

**Conclusions:**

The results of this study suggest that IL-6 is better than PCT and CRP in predicting the treatment success in predominantly non-surgical sepsis in the first 48–72 h.

## Background

The population incidence of severe Sepsis and septic shock in the industrial nations ranges between 51 and 110/100.000 and these two syndromes belong to the most frequent causes of admission to the intensive care unit (ICU). The mortality rate is still very high, with a hospital mortality rate of up to 50% [1]. The introduction of the sepsis management bundles contributed to the reduction of sepsis mortality [2].

The crucial issue in sepsis management is the prompt treatment of the underlying infection [3, 4] or surgical focus eradication. Daily evaluation of the antibiotic regimen based on clinical and microbiological criteria and a decision regarding appropriate de-escalation strategy is recommended [2]. However, because microbiological evidence of the infection is not possible in every case, the implementation of laboratory inflammation markers may be useful [5]. Procalcitonin (PCT) has been extensively investigated as a marker of sepsis [2]. It is also evaluated in a few studies regarding its merits to reduce the duration of antibiotic therapy [6–8]. A recent meta-analysis concluded that PCT-guided antibiotic treatment improves survival and reduces antibiotic exposure in critically ill patients with sepsis of any type [9]. Serum PCT levels start to rise 6–12 h after the infectious stimulus, while the maximum daily decrease under effective anti-infective therapy is 50% [10]. However, with a sensitivity of 87.6% and specificity of 73.3%, there is still a certain degree of uncertainty regarding the use of PCT as a marker of sepsis diagnosis [11].

C-reactive protein (CRP) starts to rise about 4–6 h after an inflammatory stimulus, the concentration doubles every 8 h, with a maximum concentration after 36–50 h. If the inflammatory stimulus is controlled, CRP decreases but with a half-life of 19 h. An increase in CRP of at least 22 mg/l in the first 48 h was associated with ineffective antibiotic therapy [12], while a reduction by at least 50 mg/l within 4 days was associated with recovery [13].

The relatively slow dynamics of PCT and CRP in sepsis is a relevant issue in the intensive care management of patients. Therefore, the search for other biomarkers is warranted. One candidate is the pro-inflammatory cytokine interleukin-6 (IL-6). Serum IL-6 rises within a few minutes after a stimulus (infection, trauma or any other inflammation), with a half-life of about an hour [14, 15]. The level of IL-6 correlates with the extent of the inflammation, severity of organ dysfunction and sepsis-associated death [16]. Several studies have shown that a rapid decrease in IL-6 in sepsis is associated with a better survival rate [11, 17]. However, these studies included a small and heterogeneous patient population, so that the results cannot be generalized. The aim of the present study was to evaluate the dynamics of IL-6, PCT and CRP in a large population of adult patients with sepsis regarding their timely prediction of success of antibiotic therapy and survival.

## Methods

### Patient selection

**Fig. 1:**
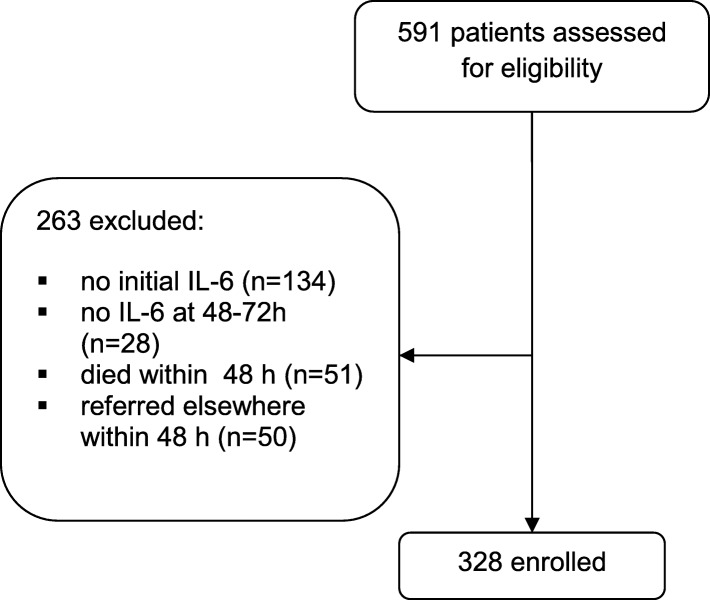
Recruitment flow chart

This is a retrospective analysis of prospectively collected data on adult patients with severe sepsis and septic shock managed at the medical ICU of the University Hospital Leipzig between September 2009 and January 2012. The routine clinical and laboratory data of patients were collected under a framework of quality control. The study was approved by the ethics commission of the University of Leipzig. Patients were included based on the definitions of Sepsis Survival Campaign 2012 [2]. A total of 591 patients with the diagnosis of severe sepsis and septic shock were identified, of whom the data quality was considered adequate for the present analysis in 328 cases (Fig. 1).

Demographic data, underlying disease conditions and the Acute Physiology And Chronic Health (APACHE) II score were documented on admission. Data for CRP, PCT, IL-6 and the Sequential Organ Function Assessement (SOFA) score on admission as well as after 24 and 48–72 h were collected. Interventions for eradication of the infection focus were also documented. Antibiotic therapy was defined as clinically successful if the patient survived the ICU stay. Because of the high mortality rate due to severe sepsis and septic shock, survival is considered to be a suitable end point of actual treatment.

### Laboratory tests

Serum PCT (ng/ml) was measured using electroluminescence immunoassay (Brahms, Berlin, Germany). CRP (mg/l) and IL-6 (pg/ml) measurements were performed using Cobas 6000 and 8000 analyzers (Roche, Mannheim, Germany) according to the manufacturer’s instructions.

### Statistical analyses

Statistical analyses were conducted using the IBM SPSS Version 20. Categorical variables were analyzed using the χ^2^ test. Numerical data were tested for their normal distribution using Kolmogorov-Smirnov test. Based on this, data with normal distribution were tested using the Student t-test, while not normally distributed data were analyzed using the Mann-Whitney U-test. Analysis of classifiers was conducted using receiver operating characteristic (ROC) curves, and the area under the curve (AUC) was calculated for IL-6, PCT and CRP regarding clinical success of antibiotic therapy. Because of high inter individual variability and logarithmic distribution of IL-6 (18, 19, 20), the quotients of the initial divided by the following values of these biomarkers regarding clinically successful antibiotic therapy were examined in the ROC analyses. Numerical data with normal distribution are presented as mean ± standard deviation, while not normally distributed data are given as median with 25 and 75% percentiles. The odds ratio (OR) with 95% confidence interval (CI) was calculated for effect estimate. The quotient of decrease in biomarker survived (DS) and decrease in biomarker died (DD) divided by the quotient of no decrease in biomarker survived (NDS) and no decrease in biomarker and died (NDD) was used in this case (OR = [DS/DD]/[NDS/NDD]). A *p* value of < 0.05 was considered statistically significant.

## Results

### Characterisation of patients

Severe sepsis was observed in 134 patients (40.9%), while 194 (59.1%) patients were suffering from septic shock at the time of ICU admission. The ICU mortality rate was 36.0% (118/328). Septic shock was more frequent among non-survivors than survivors (66.9% vs. 54.8%, *p* = 0.031). Table 1 shows baseline characteristics of the patient population classified according ICU survival. Pulmonary infections and a higher SOFA score were associated with less favourable outcome, whereas infections of the urinary tract or vascular access were associated with a better prognosis.

**Table 1:** Baseline characteristics of the patients on the day of ICU admission or diagnosis of sepsis

	Survivors (*n* = 210)	Non-survivors (*n* = 118)	p (survivors vs. non-survivors)
Median age	64 [54;73]	62 [55;69]	0.319
Males	128/210 (61.0%)	79/118 (66.9%)	0.280
Median BMI	25.0 [22;29]	24.0 [21.75;28]	0.248
APACHE II score	26.6 ± 8.1	28.0 ± 7.6	0.110
SOFA score	7.6 ± 3.9	10.1 ± 3.9	< 0.001
Sepsis focus			
Pulmonary	102/210 (48.6%)	84/118 (71.2%)	< 0.001
Urinary tract	42/210 (20.0%)	10/118 (8.5%)	0.006
Abdomen	18/210 (8.6%)	11/118 (9.3%)	0.818
Vascular access	16/210 (7.6%)	0/118 (0%)	0.002
Skin and soft tissue	6/210 (3.3%)	3/118 (1.7%)	0.867
Endocarditis	4/210 (1.9%)	2/118 (1.7%)	0.892
Others	22/210 (10.5%)	8/118 (6.8%)	0.265

*BMI* Body-Mass-Index, *APACHE II score* Acute Physiology And Chronic Health Evaluation II score, *SOFA score*, Sequential Organ Failure Assessmentscore, *IL-6* Interleukin-6, *PCT*, Procalcitonin, *CRP* C-reactive protein

Microbiological investigations were positive in 70.1% of cases, showing the following results: gram positive organisms only (18.6%), gram negative organisms only (31.4%), candida only (4.6%), mixed flora (14.9%), others (0.6%). Initial antibiotic therapy included piperacillin/tazobactam (40.9%), carbapenem (35.7%), fluorchinolone (25.0%), glycopeptide (18.6%) and an echinocandin (11.3%), either alone or in combination. Surgical focus eradication was carried out in 13.4% cases.

### Biomarkers at admission

On ICU admission, the serum concentrations for PCT and CRP were significantly higher in survivors than in non-survivors (PCT: 4.9 [1.3;24.8] vs. 2.9 [1.3;24.8] ng/ml, *p* = 0.032; CRP 165.3 [96.1;274.8] vs.149.8 [63.9;226.5] mg/dl, *p* = 0.047), while this difference was not significant, although with a trend, for IL-6 (600.6 [218.9;2743.5] vs. 381.6 [170.3;1558.0] pg/ml, *p* = 0.083).

### Kinetics of IL-6

**Fig. 2:**
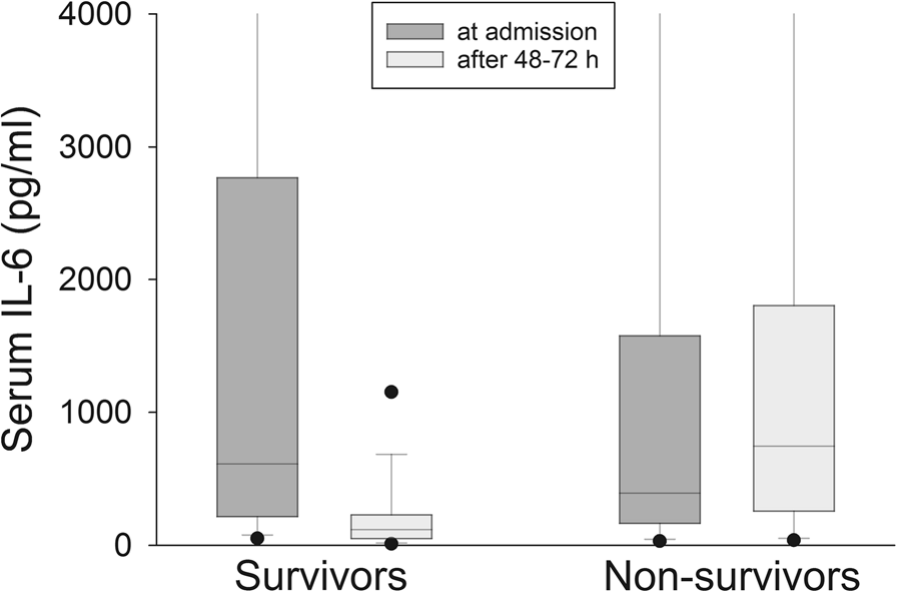
Serum IL-6 levels in survivors and non-survivors on ICU admission and after 48-72 h. Dots at the lower and upper sides of the whiskers represent 5th and 95th percentile, respectively

Sequential analysis of the dynamics of IL-6 among survivors showed that it decreased by a median factor of 1.92 [0.93;8.39] after 24 h and by a factor of 4.90 ([1.63;22.80] after 48–72 h. In contrast, IL-6 increased in non-survivors by a factor of 1.08 [0.54;2.40] after 24 h and by a factor of 1.37 [0.48;4.81] after 48–72 h. Median serum IL-6 after 48–72 h was significantly lower in survivors than in non-survivors (114.2 pg/ml [54.4;229.9] vs. 746.6 pg/ml [261.8;1808.8]; *p* < 0.001; Fig. 2).

176/224 patients (78.6%) with an IL-6 decrease after 48–72 h survived, while 70/102 (68.6%) of the patients without IL-6 reduction after 48–72 h died, which translates into a positive predictive value of 0.79 and a negative predictive value of 0.69. These results are highly significant (OR 8.02; CI 4.74–13.57).

### Kinetics of PCT

**Fig. 3:**
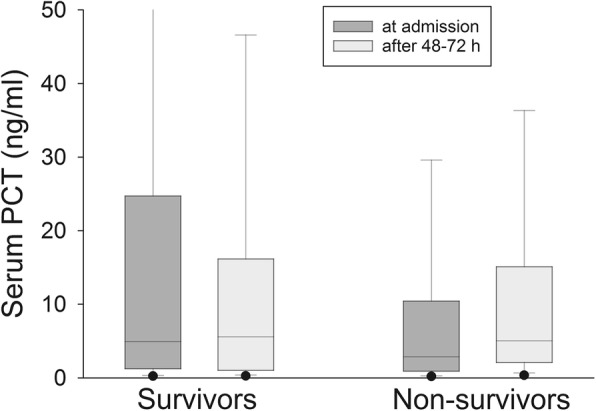
Serum PCT levels in survivors and non-survivors on ICU admission and after 48-72 h. Dots at the lower and upper sides of the whiskers represent 5th and 95th percentile, respectively

In contrast, PCT even increased in survivors after 24 h by a factor of 1.07 [0.79;1.75] while it decreased after 48–72 h by a factor of 1.43 [0.66;2.37]. In non-survivors, PCT increased by a factor of 1.33 [0.90;2.39] after 24 h and by a factor of 1.26 [0.71;3.06] after 48–72 h., Median PCT values after 48–72 h did not differ significantly in survivors and non-survivors (5.6 [1.1;16.0] vs. 4.9 [2.0;14.6] ng/ml; *p* = 0,586; Fig. 3).

125/172 (72.7%) patients with a reduction in PCT after 48–72 h survived, while 70/149 (47%) of the patients without a PCT reduction after 48–72 h died, which translates into a positive predictive value of 0.73 and a negative predictive value of 0.47. These results are highly significant (OR 2.36; CI 1.48–3.75), but less than for IL-6.

### Kinetics of CRP

**Fig. 4:**
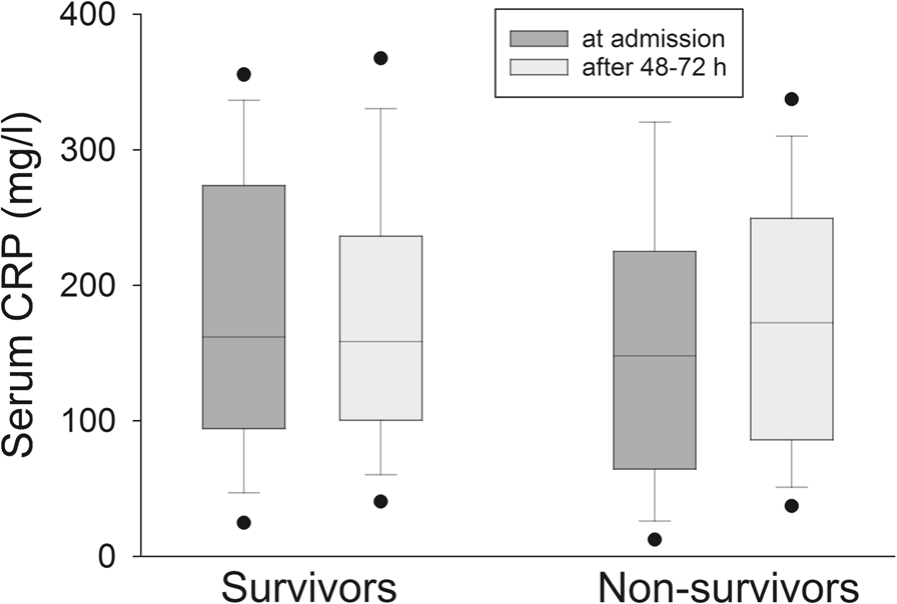
Serum CRP levels in survivors and non-survivors on ICU admission and after 48-72 h. Dots at the lower and upper sides of the whiskers represent 5th and 95th percentile, respectively

Regarding CRP, it increased in survivors by a factor of 1.09 [0.90;1.52] after 24 h, while it decreased by a factor of 1.07 [0.72;1.61] after 48–72 h. Among non-survivors, CRP increased after 24 h by a factor of 1.07 [0.92;1.40] and remained almost unchanged after 48–72 h (increase by a factor of 1.07 [0.79;1.71]).Median CRP-values after 48–72 h did not differ significantly in survivors and non-survivors (158.5 mg/l [100.5; 235.5] vs. 172.4 mg/l [86.2; 249.3]; *p* = 0.988; Fig. 4).

107/157 (68.2%) patients with a CRP reduction after 48–72 h survived, while 61/152 (40.1%) patients with no CRP reduction after 48–72 h died, resulting in a positive predictive value of 0.68 and a negative predictive value of 0.40, which is not statistically significant (OR 1.44; CI 0.90–2.29).

### ROC analyses

**Fig. 5:**
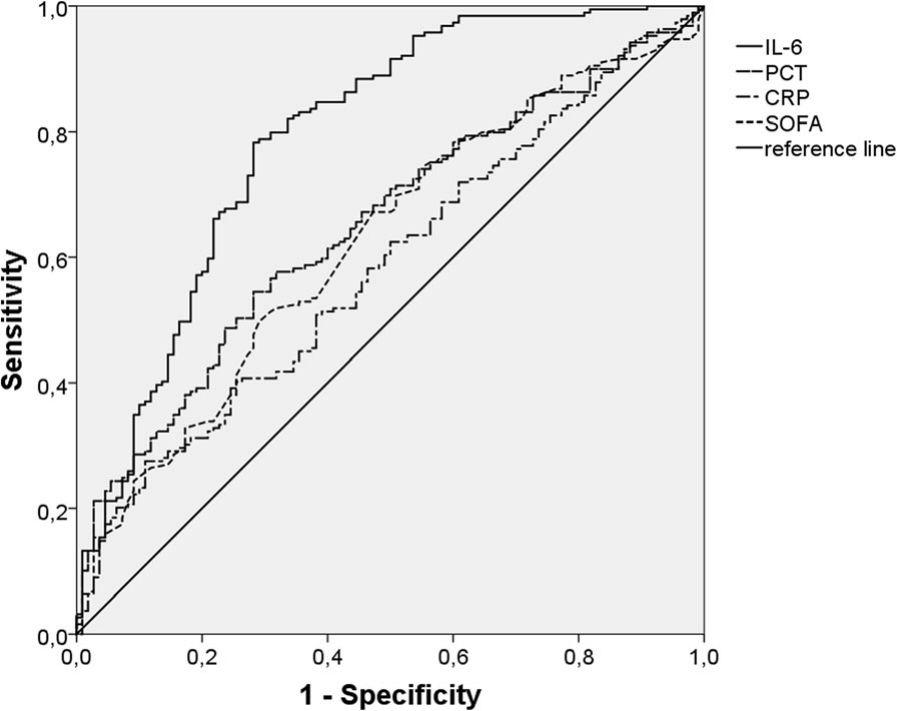
Survival ROC curves for quotient of IL-6, PCT, CRP levels and SOFA score on ICU admission divided by the corresponding values after 48-72 h

The AUC for IL-6 was higher than that for PCT and CRP after 24 h (IL-6: 0.701, *p* < 0.001; PCT: 0.594, *p* = 0.009; CRP: 0.490, *p* = 0.783) and after 48–72 h (IL-6: 0.792, *p* < 0.001; PCT: 0.650, *p* < 0.001; CRP: 0.584, *p* = 0.015). As a comparison, the AUC for the SOFA score was 0.557 (*p* = 0.115) after 24 h and 0.624 (*p* < 0.001) after 48–72 h (Fig. 5).

## Discussion

This study showed a significant reduction in serum IL-6 in survivors already one day and strongly pronounced two to three days after ICU admission. PCT was much weaker in predicting a successful therapy after 48–72 h. The dynamics of serum CRP in the same period did not help predict successful therapy. In a small prospective study, Jekarl et al. reported that survivors of sepsis showed a rapid IL-6 reduction, while non-survivors showed persistently high IL-6 concentrations [11]. These findings could be confirmed by Oda et al. [17] and Pallás Beneyto et al. [18]. In another investigation in 24 patients with severe sepsis, survivors showed lower IL-6 levels, but no differences in IL-6 decline compared with non-survivors [19]. Due to its fast induction and very short half-life [14], IL-6 seems to be better than PCT and CRP to display the extent of the inflammation and treatment success.

In a prospective study on patients with postoperative sepsis, differences for the three markers of inflammation were detected first on day 7 that allowed prediction of 28-day survival [20]. The authors did not observe any significant difference between IL-6, PCT and CRP regarding mortality prediction in the early phase of the inflammation. The additional surgical trauma in their patient population may have had a relevant influence on the dynamics of the inflammatory markers. In contrast, surgical eradication of the infection focus was required in only 13.4% of our patient population.

Ruiz-Rodriguez et al. showed in a cohort of patients with septic shock that the PCT clearance after 24 and 48 h was significantly better in survivors than non-survivors [21]. Although their findings are similar to ours, this effect was not as good as that with IL-6. The strength of PCT seems to be in diagnosing sepsis [22–24] and probably in managing the duration of antibiotic therapy [9].

Meisner et al. reported that the course of both PCT and CRP in the first four days did not allow any discrimination between survivors and non-survivors of sepsis or systemic inflammatory response syndrome (SIRS) [25]. In another prospective study in 50 septic patients, a CRP increase of 22 mg/l after 48 h was associated with an ineffective antibiotic therapy, however with a limited sensitivity and specificity [12]. In our study, CRP was not predictive after 24 as well as 48–72 h. The slow kinetics of CRP is probably the reason for this observation. On the other hand, a high CRP on discharge from the ICU correlates with a poor prognosis during further clinical course [26].

A recent prospective observational study in 50 septic patients demonstrated a significant PCT and IL-6 decline at day 5 in survivors compared with non-survivors. Nevertheless, these findings could not be confirmed at day 2 like in our investigation [27].

The SOFA score showed in our study a significant reduction in survivors after 48–72 h, which is similar to the observation by Jones et al. [28]. However, the AUC for IL-6 was better than that for the SOFA score in our study. This observation is most probably not surprising, since control of inflammation should ensue earlier than recovery of organ function.

Interestingly, survivors showed at admission significantly higher PCT and CRP levels as well as a trend towards higher IL-6 levels than non-survivors. Literature data in this regard are not homogenous [17, 19, 20, 24, 27, 29].

The strength of our study is the systematic documentation of a large and well characterized cohort of patients with sepsis with a constant team of care givers. Its limitations are that it is a monocentric and retrospective analysis. Furthermore, the analysis was carried out in mostly non-surgical septic patients, so that our observations cannot be easily extrapolated to other critically ill patients. Because of the large inter-individual variation in the induction of IL-6, the quotient between the initial and the control levels of IL-6 was used and compared with the quotients for PCT and CRP. The variance for PCT and CRP is smaller, so that the difference between the initial and the control values may be suitable for prediction of prognosis [12, 21]. However, computing a quotient for these biomarkers allows a better use of them in daily clinical practice.

## Conclusions

In conclusion, our results suggest that IL-6 is better than PCT and CRP to predict the treatment success of non-surgical sepsis within the first 48–72 h. The role of early interventions, re-evaluation of septic focus or changing antibiotic therapy in case of lack of significant reduction in IL-6 should be investigated prospectively.

## Abbreviations

APACHE II: Acute Physiology And Chronic Health Evaluation II
AUC: area under the curve
BMI: Body-Mass-Index
CI: confidence interval
CRP: C-reactive protein
DD: decrease died
DS: decrease survived
ICU: intensive care unit
IL-6: interleukin-6
NDD: no decrease died
NDS: no decrease survived
OR: odds ratio
PCT: procalcitonin
ROC: receiver operating characteristics
SOFA-Score: Sequential Organ Failure Assessment-Score
